# A Recipe for Resilience: A Systematic Review of Diet and Adolescent Mental Health

**DOI:** 10.3390/nu17233677

**Published:** 2025-11-24

**Authors:** Jade E. Tucker, Anthony M. Brennan, David Benton, Hayley A. Young

**Affiliations:** Faculty of Medicine, Health and Life Science, Swansea University, Swansea SA2 8PP, UK; 1905351@swansea.ac.uk (J.E.T.); a.m.brennan@swansea.ac.uk (A.M.B.);

**Keywords:** adolescents, mental health, depression, anxiety, diet quality, dietary patterns, micronutrients, systematic review

## Abstract

**Background/Objectives:** Adolescence is a critical period of vulnerability for the onset of mental health difficulties, presenting an urgent need for scalable prevention strategies. Diet is a universal, modifiable factor, yet its evidence base remains inconsistent. This systematic review synthesised evidence from controlled trials and prospective cohort studies investigating the relationship between diet and mental health in adolescents aged 10–19 years. **Methods:** Searches were conducted to 20 July 2025, and risk of bias was assessed. **Results:** Nineteen studies met the inclusion criteria: six intervention trials and thirteen cohort studies. Examined exposures included vitamin D, omega-3s, polyphenol-rich foods, Mediterranean-style diets, and overall diet quality. Depressive symptoms were the most studied outcome, though the synthesis also included other dimensional outcomes such as anxiety, stress, well-being, and internalising/externalising indices. Across designs, healthier dietary patterns were often associated with fewer depressive symptoms, while poorer diet quality was linked to increased psychological distress. However, the current evidence is constrained by wide variation in assessments, small samples, and significant methodological limitations—particularly with high risk or some concerns noted in half of the included intervention trials—along with evidence suggesting that associations may differ by sex and are often sensitive to adjustment for socioeconomic status. **Conclusions:** Despite these challenges, the findings suggest diet as a possible, actionable target for supporting adolescent mental health. This review concludes by proposing a detailed roadmap for future research, prioritising harmonised symptom-based outcomes, biomarker-verified assessments, explicit analysis of sex and socioeconomic (SES) effects, and adequately powered trials to inform effective public health strategies for youth. Protocols were registered with PROSPERO (CRD42023413970) and archived on the Open Science Framework.

## 1. Introduction

Adolescence is a critical developmental window characterised by rapid social, psychological, and biological change. It is also a period of heightened vulnerability, with early symptoms of mental health difficulties emerging as early as ages 7–11 [1]. Globally, around one in five adolescents experience mental health problems, underscoring the need to identify modifiable risk and protective factors. Within this context, diet has gained increasing attention as a plausible, scalable influence on adolescent mental health [2].

Building on this, the consequences of early onset mental health problems are profound. Difficulties that emerge in adolescence frequently persist into adulthood, elevating risk for recurrent depression and anxiety, poorer academic attainment [3], impaired social functioning, and reduced quality of life [4]; see also [5]. These sequelae can derail key developmental milestones and contribute to the intergenerational transmission of risk. This developmental period therefore represents a critical opportunity for prevention and early intervention. Within this context, diet has been highlighted as a modifiable, scalable factor embedded in daily life; however, the evidence base remains heterogeneous and at times inconsistent, motivating a systematic synthesis.

In parallel, traditional interventions, e.g., psychotherapy and pharmacotherapy, are available but insufficient to meet the rising burden of adolescent mental health difficulties. These approaches can be costly, carry side-effects, and remain inaccessible for many, particularly in low-resource settings [6]. They also rarely address lifestyle and environmental determinants that may underlie psychological vulnerability. In contrast, dietary patterns represent a potential promising, scalable target with direct relevance to adolescents—who are simultaneously gaining autonomy over food choices, facing heightened nutritional demands for growth and brain development, and showing susceptibility to unhealthy behaviours that can track into adulthood [7,8]. These developmental features provide a strong rationale for considering diet as a leverage point in adolescent mental health.

Taken together, the evidence supports the plausibility of a diet–mental health relationship in adolescence. Epidemiological studies consistently associate poorer diet quality with greater depressive and anxiety symptoms, whereas healthier dietary patterns are linked to more favourable outcomes [9,10]. Multiple pathways provide biological credibility—including diet-related modulation of inflammation, oxidative stress, gut microbiota, and neurotrophic factors—all implicated in mental health [11]. However, much of the direct evidence for these specific pathways is derived from adult or pre-clinical models, and their precise role within the unique developmental context of adolescence remains a critical area for investigation. Intervention studies—spanning nutrient supplementation and whole-diet modification—report mixed effects, with considerable variability in design, dietary assessment, outcome measures, and effect sizes as well as differing risks of bias. Collectively, these limitations constrain causal inference and the strength of current conclusions.

Prior syntheses in youth have typically narrowed either the exposure or the outcome, for example, reviews centred on supplementation (omega-3 or vitamin D) in children and adolescents with depression, and diet-as-treatment RCTs targeting depressive symptoms specifically [12]. Likewise, several adolescent reviews restrict outcomes to depression, linking diet quality or healthy patterns with fewer depressive symptoms but offering limited coverage of broader mental-health endpoints [13]. Many also organise evidence by diagnostic categories, which can obscure symptom heterogeneity, underrepresent subclinical distress, and reduce comparability across tools and settings [14,15]. Many have also overlooked the potential influence of sex and socioeconomic status. Therefore, the aim of this systematic review was to synthesise and evaluate the most rigorous evidence (randomised controlled trials and longitudinal cohorts) and consider a wider range of dimensional outcomes including depressive and anxiety symptoms, stress, well-being, and internalising/externalising indices. Crucially, we also synthesised available evidence regarding the influence of sex and socioeconomic status on these diet–mental health relationships. This comprehensive approach aims to improve construct coverage and the real-world relevance of findings beyond clinic-defined groups, thereby enhancing applicability for both clinical practice and public-health policy.

## 2. Materials and Methods

This review was conducted in accordance with the Preferred Reporting Items for Systematic Reviews and Meta-Analyses (PRISMA 2020) guidelines [16]. Protocols were registered with PROSPERO (CRD42023413970) and archived on the Open Science Framework (https://osf.io/c6xze (accessed on 20 October 2025)). PRISMA 2020 checklists are provided (Prisma Checklist S1a,b from Supplementary Materials). The review proceeded in two phases. In Phase 1, literature searches were conducted to scope the field using preliminary eligibility criteria. In Phase 2, final research questions and eligibility criteria were applied.

### 2.1. Search Strategy and Selection Criteria

A systematic search of Scopus, PubMed, and PsycINFO was conducted up to 20 July 2025 to identify relevant literature published in English. The search strategy combined three core concepts: (1) an adolescent population (e.g., “Adolescents” OR “Adolescence”); (2) nutrition-related exposures (e.g., “Food” OR “Meal” OR “Diet”); and (3) mental health outcomes (e.g., “Mental health” OR “Mood” OR “Depression”). The search was further refined to include only prospective study designs (e.g., longitudinal, cohort, RCT) and exclude cross-sectional studies, review articles, and studies focused on eating disorders. To identify unpublished studies, we also searched the British Library of Electronic Theses Online Service (EThOS). A full, unabridged example of the search string used for Scopus is available in the Supplementary Materials. The strategy was implemented by H.Y. and J.T. Reference lists of included studies and relevant reviews were hand-searched.

**Figure 1:**
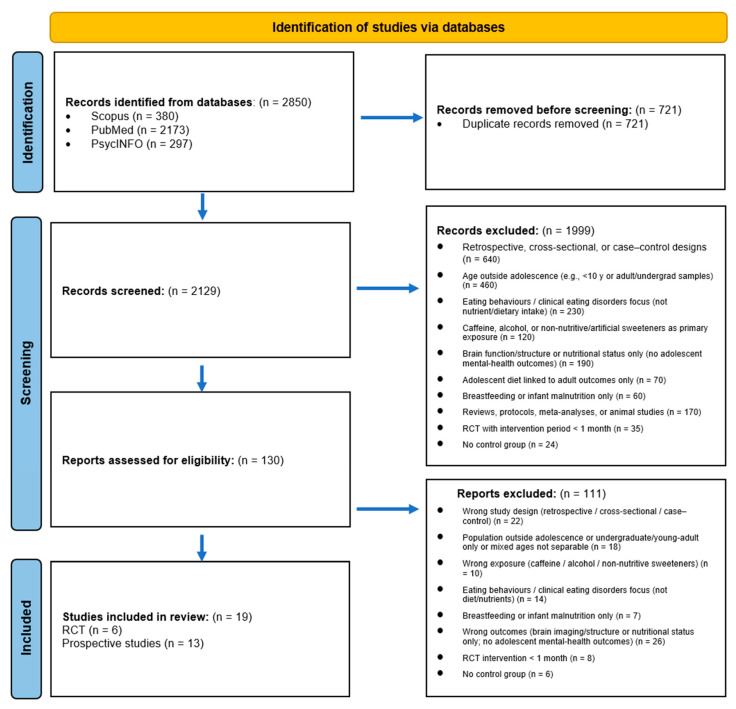
PRISMA flow diagram of the study selection process.

Study selection. Records were de-duplicated and screened independently by the two reviewers (titles/abstracts, then full texts). Disagreements at each stage were resolved through iterative discussion to achieve consensus. Where consensus could not be reached, a third reviewer (A.B.) acted as an arbiter. Quantitative inter-rater agreement (e.g., Cohen’s kappa) was not calculated, as this consensus-based process was used to ensure consistency. Reasons for full-text exclusion were recorded. The selection process is shown in the PRISMA flow diagram (Figure 1).

### 2.2. Eligibility Criteria

#### 2.2.1. Inclusion

Eligible studies enrolled adolescents aged 10–19 years (World Health Organisation definition) drawn from the general population, using the National Academies of Sciences, Engineering, and Medicine definition (i.e., including individuals with, or at risk for, chronic disease such as overweight/obesity unless a nutrient-specific exception applied), or adolescents with nutritional deficiencies. Eligible designs comprised randomised controlled trials and prospective cohort studies, including cluster variants; acute single-consumption trials were excluded, and chronic interventions were defined as lasting one month or longer. Eligible exposures included dietary patterns or nutrient intake assessed during adolescence. Outcomes were required to be assessed using validated mental-health instruments. Only full-text, peer-reviewed articles published in English were considered.

#### 2.2.2. Exclusion

Exclusions were: animal studies, reviews, protocols, and meta-analyses; samples outside adolescence (e.g., <10 years or exclusively undergraduate/young-adult populations); studies focused on eating behaviours or clinical eating disorders rather than nutrient/dietary intake; studies examining caffeine, alcohol, or non-nutritive/artificial sweeteners as the primary exposure (sugar-sweetened beverages were eligible); studies reporting only brain function/structure or nutritional status without adolescent mental health outcomes; studies relating adolescent diet to adult outcomes only; studies focused solely on breastfeeding or malnutrition in infancy; and retrospective, cross-sectional, or case–control designs.

### 2.3. Data Extraction

Data were extracted by a single reviewer (J.T.) using a piloted template and independently verified by a second reviewer (H.Y.) for completeness and accuracy. Disagreements were reconciled by a third author (A.B.). Extracted items included: author and year; country and study setting; exposure definition (dietary intervention and duration for trials; dietary assessment method for cohorts); sample size; participant characteristics (mean age and standard deviation, BMI where reported, sex/gender distribution, location, baseline health status); study design; mental-health measures (instrument and timing); primary outcomes with effect estimates and precision; covariates/adjustment strategy; funding/source of support; and reviewer notes on interpretation. When the required information was unclear or missing, the study authors were contacted for clarification.

### 2.4. Outcome Measures

The primary outcome measure was mental health, assessed using validated and standardised tools such as mood and depression scales. Secondary outcome measures included self-reported or clinically observed indicators of psychological well-being, emotional regulation, and behavioural health. Additional outcomes comprised objective indicators of nutritional status, dietary intake, or relevant biomarkers, provided these were examined in relation to changes in mental health or psychological outcomes.

### 2.5. Synthesis and Organisation Process

Given the wide heterogeneity observed in the study designs, dietary exposures, and specific outcome measures (e.g., different scales for depression), a formal statistical meta-analysis was not deemed appropriate. Therefore, we conducted a narrative synthesis of the findings. Studies were organised first by design (randomised controlled trials vs. prospective cohort studies) and then by dietary exposure. For trials, categories reflected the intervention type (e.g., vitamin D, omega-3 fatty acids, polyphenol-rich foods such as wild blueberries, multi-component whole-diet modification). For prospective cohorts, categories reflected the exposure assessed (e.g., adherence to Mediterranean-style patterns, macro-/micronutrient intake, sugar-sweetened/soft-drink consumption, overall diet quality indices). Results are presented in this hierarchy to maintain comparability within design and exposure type.

### 2.6. Risk of Bias

Risk of bias was assessed using design-appropriate, established tools. For randomised controlled trials, the Cochrane Risk of Bias 2 (RoB 2) tool was applied [17], with cluster adaptations used where applicable. For prospective cohort studies, the Joanna Briggs Institute Critical Appraisal Checklist for Cohort Studies was used. All assessments were conducted after data extraction by two independent reviewers (H.Y. and J.T.); disagreements were resolved by discussion or, where necessary, consultation with a third reviewer (A.B.). Additional information was sought from study protocols or by contacting the authors when possible. Domain-level judgements and overall ratings (low/some concerns/high for RoB 2; low/moderate/high for JBI) are reported in Supplementary Tables S1 and S2.

## 3. Results

### 3.1. Study Selection

The PRISMA flow diagram is shown in Figure 1. In total, 2850 records were identified across databases (Scopus = 380; PubMed = 2173; PsycINFO = 297). After de-duplication (n = 721), 2129 titles/abstracts were screened and 1999 were excluded. A total of 130 full texts were assessed for eligibility, of which 111 were excluded for pre-specified reasons (e.g., wrong design, population, exposure, or outcome; insufficient data), leaving 19 studies for inclusion: six randomised controlled trials and thirteen prospective cohort studies. Full-text exclusion reasons are summarised in Figure 1.

### 3.2. Randomised Controlled Trials (RCTs)

Six RCTs evaluated dietary interventions in adolescents: vitamin D supplementation [18,19,20], omega-3 fatty acids [21,22], and a polyphenol-rich whole food (wild blueberries [23]). Samples were predominantly mixed-sex, and participants were generally healthy. Adolescent mental health outcomes were assessed with validated instruments (Table 1).

Depressive symptoms were the most frequently evaluated endpoint, appearing in four trials [18,20,21,23]. Measures included the Beck Depression Inventory [20], the Mood and Feelings Questionnaire [23], the Centre for Epidemiologic Studies Depression Scale [21], and YSR/CBCL internalising subscales [18]. Aggressive or externalising behaviours were assessed in one trial [18]. Self-esteem was examined once using the Rosenberg Self-Esteem Scale [21]. Anxiety-related outcomes featured in two trials: one reported RCADS anxiety risk [23], and the other reported DASS anxiety/stress together with WHOQOL-BREF domains [19]. The Isaac et al. trial did not report depression scores as an outcome; in contrast, the Fisk et al. trial reported depression as a primary outcome, measured separately with the MFQ. Transient affect was assessed using PANAS-Now [23] and a 10-item visual analogue mood scale [22].

Across these interventions, effects on mental-health endpoints were heterogeneous. Favourable effects were seen in some but not all trials that used high-dose supplementation or targeted populations with potentially low baseline nutrient status [20]. In contrast, some shorter or lower-dose interventions and one long-term trial hampered by poor adherence yielded null findings [19,21,22]. Interpretation was further constrained by variation in outcome selection (multiple instruments tapping overlapping constructs), small samples, and variation in adherence or biomarker verification, which together limited comparability across trials. Full intervention characteristics and measurement instruments are summarised in Table 1.

#### 3.2.1. Vitamin D Supplementation

Three RCTs evaluated vitamin D supplementation. Trials lasted three months [18,19] and were conducted in Norway [18], and India [19,20]. Dosing regimens varied markedly: one trial provided 38 µg/day (≈1520 IU) versus the placebo to 50 adolescents [18]; a school-based study delivered a fortified snack containing 1000 IU vitamin D_3_/day to 71 adolescents [19]; and a cluster-randomised trial provided an effective daily dose of 2250 IU while the controls received 250 IU plus calcium [20]. Across the three trials, the signal for mental health benefits was mixed. In rural India, a cluster RCT also reported a small-to-moderate reduction in depressive symptoms (mean difference ≈ −3.4 BDI-II points), which was consistent across analyses [20]. In contrast, the snack-fortification trial in India found no change in health-related quality of life (WHOQOL-BREF) or anxiety and stress (DASS-21) [19]. Similarly, the trial in Norway noted that while supplementation corrected vitamin D deficiency, it did not alter the internalising or externalising outcomes (YSR-CBCL), although an exploratory analysis suggested a link between low baseline vitamin D and greater externalising problems [18].

In sum, based on the three included RCTs, there is emerging but inconsistent evidence that vitamin D supplementation may reduce depressive symptoms in adolescents. This effect was most apparent in the largest trial, which used a relatively higher dose [20], while the two smaller trials using lower doses reported null findings for their primary mental health outcomes. There was minimal evidence from this set of studies for benefits on externalising behaviour, anxiety, stress, or quality of life [18,19]. Confirmation in larger, rigorously designed RCTs—ideally with baseline status stratification, biomarker verification, and standardised outcome batteries—is warranted.

#### 3.2.2. Fatty Acids

Two trials evaluated omega-3 fatty acid supplementation, with intervention durations ranging from 8 weeks (UK [22]) to 12 months (The Netherlands [21]). Although both targeted omega-3, the procedures and implementation differed substantially.

In Kennedy et al. [22], 90 healthy 10–12-year-olds were randomised to the placebo, 400 mg/day DHA (two capsules morning, placebo evening), or 1000 mg/day DHA (capsules morning and evening); capsules also contained 4 mg EPA and vegetable oil. Compliance exceeded 80% under parental supervision. Outcomes comprised computerised cognitive tasks and a visual-analogue mood scale. Apart from a single effect on relaxation favouring both DHA arms, no consistent benefits for cognition or broader mood were detected, plausibly reflecting the short exposure period and modest per-arm sample sizes (n ≈ 30).

Van der Wurff et al. [21] conducted a double-blind, placebo-controlled trial (n = 256; age 14–15 years) with krill-oil EPA + DHA. Cohort I escalated from 400 mg/day (260 mg EPA, 140 mg DHA) for three months to 800 mg/day (520 mg EPA, 280 mg DHA); Cohort II commenced at the higher dose. Placebo oils resembled a typical European fatty-acid profile. Adherence was poor: by six months, the mean Omega-3 Index (O-3I) in the active group reached only 5.29% (target 8–11%), declining to 4.86% at twelve months, indicating missed doses on ~44% of days between months 6–12. Primary outcomes were depressive symptoms (CES-D) and self-esteem (Rosenberg). Neither intention-to-treat nor O-3I-based analyses showed significant intervention effects. At 12 months, depressive symptoms trended non-significantly lower in the active group (M = 10.86, SD = 8.92) versus the placebo (M = 13.60, SD = 11.95; *p* = 0.066; d = 0.20), and self-esteem was marginally higher (M = 23.00, SD = 4.96 vs. 21.74, SD = 6.36; *p* = 0.120; d = 0.15).

Taken together, the current data are not sufficient to support a robust effect of omega-3 supplementation on adolescent mood outcomes. A short, well-adhered DHA trial yielded at most a modest, isolated improvement in subjective relaxation [22] (2009), whereas a longer trial with suboptimal adherence and sub-target O-3I showed a borderline benefit for depressive symptoms or self-esteem [21]. Future trials should prioritise adequate duration, dose, and exposure verification (e.g., O-3I targets) with sufficient power to detect clinically relevant effects and test the baseline-status moderation.

#### 3.2.3. Polyphenols/Wild Blueberry

A four-week, double-blind, placebo-controlled RCT in the United Kingdom [23] examined daily wild-blueberry (WBB) supplementation in adolescents aged 12–17 years (n = 64). Participants consumed either a flavonoid-rich WBB drink delivering ~253 mg anthocyanins/day or a placebo matched for sugars and vitamin C. Baseline depression and anxiety were subclinical in both groups. After four weeks, the WBB group reported lower depressive symptoms than the placebo (Cohen’s *d* = 0.65), with no between-group differences for anxiety or positive/negative affect. Although limited by short duration and modest sample size, this study provides preliminary evidence that short-term flavonoid supplementation may reduce self-reported depressive symptoms in adolescents [23]. Replication in larger samples, including clinically elevated cohorts, is warranted.

#### 3.2.4. Summary of Randomised Controlled Trials

Across six RCTs, interventions targeted vitamin D, omega-3 fatty acids, and a polyphenol-rich whole food (wild blueberries), with outcomes spanning depressive symptoms, anxiety, self-esteem, externalising behaviours, and transient mood. The clearest signal concerned depressive symptoms. One larger vitamin D trial reported small-to-moderate improvements [20], while two smaller trials reported no effect on their primary mental health outcomes [18,19]. The single trial of polyphenols (wild blueberries) also reported a positive effect on depressive symptoms [23], whereas the two omega-3 trials yielded null findings for mood [21,22]. Attrition was a notable limitation in several studies, with rates exceeding 20% in some trials [20,21]. Furthermore, short exposure periods [22,23] limited sample sizes in some studies [18,19,22,23], heterogeneity in outcome instruments, and suboptimal adherence [21] constrained inference. Larger, longer, and methodologically rigorous trials—ideally stratified by baseline nutrient status and employing standardised outcome batteries—are required to clarify the efficacy of these interventions for adolescent mental health.

### 3.3. Prospective Studies

The review’s search identified thirteen prospective cohort studies. Unlike RCTs, these followed the participants longitudinally without intervention, evaluating associations between habitual dietary exposures and subsequent adolescent mental health. Exposures encompassed Mediterranean-style adherence [24,25,26], macronutrient intake [27,28], micronutrient intake [29,30], soft-drink consumption [31], and overall dietary pattern/quality indices [32,33,34,35,36]. Most studies recruited mixed-sex community samples; one focused on elite athletes [27].

Outcomes covered multiple domains of adolescent mental health. Depressive symptoms or emotional functioning were the most frequently examined endpoints [26,27,28,30,31,32,33,34] using a range of self-report instruments such as the PedsQL, BDI-Y, MFQ, PHQ-9, and a brief subscale from the DISC. Anxiety and emotional symptoms were assessed with the SCARED [24], while broader externalising and aggressive behaviours were measured using the YSR [29,35] and a dedicated aggression scale [31]. One study used the validated Flourishing Scale to assess psychological well-being, capturing positive dimensions of positive functioning such as purpose, optimism, and social connectedness [36].

Across cohorts, findings were generally consistent for dietary patterns, where healthier patterns predicted better mental health outcomes [32,33,34,35,36], although one well-controlled study found no prospective association after full adjustment for covariates [26]. For specific exposures, higher soft-drink consumption was prospectively linked to increased aggression [31], while associations for individual nutrients were varied but generally pointed towards a protective effect, including for protein [27], magnesium [29], vitamin D_3_ [30], and dietary fibre (though this effect was not independent of the overall dietary pattern) [28]. Overall, while cohort evidence supports a plausible relationship between diet quality and adolescent mental health, comparability is limited by non-aligned dietary indices and outcome instruments, and by residual confounding typical of observational designs.

#### 3.3.1. Adherence to Mediterranean Diet (MD)

Three longitudinal studies examined MD adherence in relation to adolescent psychological outcomes, but differed in direction of analysis, outcome scope, and measurement approach, yielding mixed conclusions. In Spain, a school-based cohort (n = 165; mean age 13.5 years) assessed MD adherence using the Krece Plus index (0–11) alongside FFQ-derived dietary patterns and constructed a composite “emotional symptoms” indicator from the SCARED, Children’s Depression Inventory, and Youth’s Inventory-4. Among females, emotional symptoms were associated with lower subsequent MD adherence and a greater likelihood of a sweet/fatty pattern; no associations were observed in males [24]. Notably, the analytic emphasis was on emotional symptoms predicting later diet, rather than diet predicting later symptoms. In Lebanon, a 12-month cohort (n = 563; assessments at baseline, 6, and 12 months) tracked MD adherence with the KIDMED index (0–12) and evaluated self-efficacy and academic achievement. Rising MD adherence predicted higher academic achievement at both follow-ups, and self-efficacy independently predicted achievement at 12 months; however, no clinical symptom measures were included, limiting inferences for mental-health endpoints per se [25]. Attrition and reliance on self-report warrant caution.

In the United Kingdom, the ROOTS cohort (n = 603; mean age 14.5 to 17.5 years) derived an MD score from a 4-day diet diary and examined depressive symptoms using the MFQ at baseline and follow-up. While higher baseline fruit and vegetable intake correlated with lower concurrent MFQ scores, these associations attenuated after adjustment for smoking, alcohol, and energy intake; neither the MD score nor specific food groups prospectively predicted depressive symptoms after full adjustment [26].

Taken together, longitudinal evidence linking MD adherence to adolescent mental health outcomes is limited and inconsistent. One study suggested that emotional symptoms may precede lower subsequent MD adherence [24], a second linked MD adherence to academic (not clinical) outcomes with a socio-cognitive correlate (self-efficacy) [25], and a third found no prospective association between MD adherence and depressive symptoms after robust adjustment [26]. Variability in dietary indices (KIDMED, Krece Plus, diary-based MD score), outcome definitions, and confounder adjustment, together with attrition and regional sampling, constrains comparability and weakens causal inference for MD adherence and adolescent mental health.

#### 3.3.2. Macronutrient Intake

Two prospective cohorts examined macronutrient intake in relation to adolescent depressive symptoms, focusing on distinct populations and exposures. In Swiss elite athletes, a 10-month longitudinal study (final analytic n = 79; 62% male; mean age 16.4 years) assessed diet via a 3-day recall and depressive symptoms with the PHQ-9 at baseline and follow-up [27]. Hierarchical regressions adjusting for baseline symptoms and sex indicated that higher protein intake prospectively predicted lower depressive symptom severity, whereas total fat, polyunsaturated fatty acids, carbohydrate, and sugar were not associated. Relative to the DACH reference values, the athletes’ energy, carbohydrate, and PUFA intakes were below the recommendations and sugar intake exceeded the recommendations, with protein adequate. Interpretation is constrained by the modest sample, 3-day recall, and the specificity of an elite-sport cohort.

In a general-population sample from the Western Australian Pregnancy Cohort (Raine) Study, dietary intake was captured with a 212-item FFQ at ages 14 and 17 years, with fibre intake categorised into quartiles; depressive symptoms were measured using the BDI-Y and converted to T-scores [28]. Mixed-effects logistic models adjusting for demographics, energy intake, adiposity, lifestyle, and family factors showed that adolescents in the highest fibre quartile had lower odds of moderate/extreme depressive symptoms than those in the lowest. This association persisted after excluding participants with baseline symptoms and after adjustment for hs-CRP but attenuated when overall dietary patterns (Healthy vs. Western) were included, suggesting that fibre’s apparent protective effect may be partly attributable to broader diet quality rather than an independent nutrient effect. Limitations include attrition between assessments and reliance on self-reported diet (Table 2).

Taken together, cohort evidence for macronutrients is selectively supportive. Protein intake predicted fewer depressive symptoms in elite athletes, and higher fibre intake related to lower odds of depressive symptoms in community adolescents, though the latter appeared sensitive to overall diet quality. These findings highlight the need for studies with objective or repeated dietary measures, broader biomarker panels, and samples representative beyond specialised subgroups to clarify whether macronutrient–mood associations are independent of dietary patterns.

#### 3.3.3. Micronutrient Intake

Two longitudinal cohorts examined micronutrients in relation to adolescent mental health, conducted in Australia and the United Kingdom (Table 2). Although the exposures, outcomes, and developmental windows differed, both studies provide insight into nutrient-specific associations with externalising and internalising symptoms.

In the Western Australian Pregnancy Cohort (Raine) Study, 684 adolescents were assessed at ages 14 and 17 years [29]. Dietary zinc and magnesium intake were estimated via a semi-quantitative FFQ, and externalising behaviours were measured with the Youth Self-Report (YSR). After adjustment for energy intake and relevant confounders, higher magnesium intake was associated with fewer externalising behaviours at both ages. Zinc showed a similar inverse trend that did not reach statistical significance. Interpretation is tempered by attrition between follow-ups, reliance on self-reported diet, and potential residual confounding.

Using the ALSPAC cohort, Tolppanen, Sayers [30] investigated whether childhood serum 25-hydroxyvitamin D predicted later depressive symptoms. Blood samples were obtained at a mean age of 9.8 years, and depressive symptoms were assessed with the MFQ at 10.6 years (n = 2759) and 13.8 years (n = 2752). Higher 25(OH)D_3_ concentrations predicted lower depressive symptoms at age 13.8 (adjusted odds ratio per doubling of 25(OH)D_3_ = 0.90, 95% CI 0.86–0.95), but not at age 10.6; 25(OH)D_2_ showed no association at either time point. Higher 25(OH)D_3_ was also associated with a greater likelihood of symptom improvement between 10.6 and 13.8 years. Limitations include substantial attrition, possible confounding by outdoor activity and related behaviours, and reliance on a single serum measurement.

Taken together, micronutrient findings are nutrient- and outcome-specific: magnesium intake relates to externalising behaviours, and vitamin D_3_ status (but not D_2_) relates to depressive symptoms in early-to-mid adolescence, with weaker evidence for zinc. Replication in larger cohorts with repeated dietary/biomarker assessments and comprehensive confounder control is needed to clarify directionality and generalisability.

#### 3.3.4. Soft Drink Consumption

A single longitudinal cohort examined soft-drink intake in relation to adolescent mental health, modelling reciprocal associations with aggression and depressive symptoms across three waves spanning five years [31]. Drawing on the Healthy Passages cohort (n = 5147; 51% female; mean age 11.1 years at baseline) with assessments at ages 11, 13, and 16 years, adolescents self-reported soft-drink consumption over the past seven days, aggressive behaviour (Forms and Functions of Aggression), and depressive symptoms (six items from the DISC Predictive Scales). Analyses employed an autoregressive cross-lagged model adjusting for sociodemographic, health, and behavioural covariates (including BMI, overall diet, exercise, substance use, and family factors). Cross-sectionally, higher soft-drink intake correlated with greater aggression at all three ages and with more depressive symptoms at ages 11 and 13. Longitudinally, intake at 11 predicted greater aggression at 13, and intake at 13 predicted greater aggression at 16. Aggression at 13 also predicted higher intake at 16, indicating bidirectional links for aggression. In contrast, no prospective association emerged between soft-drink intake and depressive symptoms; if anything, intake at 13 predicted slightly fewer depressive symptoms at 16. Depressive symptoms did not predict later intake.

The study’s strengths include a large, multi-site, ethnically diverse sample and pre-registered longitudinal modelling; however, exclusive reliance on adolescent self-report for both exposure and outcomes raises the possibility of shared-method variance and correlated error, which may inflate associations. Future work would benefit from objective intake measures (e.g., purchase data, biomarkers) and multi-informant mental-health assessments to reduce common-method bias and strengthen causal interpretation.

#### 3.3.5. Quality of Dietary Patterns

Six longitudinal cohorts examined overall dietary quality in relation to adolescent mental health [26,32,33,34,35,36]. Three were conducted in Australia, one in Canada [36], and two were from the United Kingdom [26,32]. Participants spanned early to late adolescence (11–18 years). Diet was assessed using FFQs, diet diaries, or composite diet-quality indices; outcomes included general mental health, depressive symptoms, anxiety symptoms, psychological well-being, and internalising/externalising behaviours.

In an Australian school-based sample (n ≈ 3000), higher healthy diet scores predicted better emotional functioning on the PedsQL, and within-person changes in diet quality over two years paralleled changes in mental health, with no evidence for reverse causation [33]. In a socially deprived UK cohort (n ≈ 2800), an unhealthy diet was cross-sectionally associated with greater odds of SDQ-defined problems (highest quintile OR = 2.10, 95% CI 1.38–3.20). Prospective analyses suggested a similar link, although effects attenuated after full adjustment for confounders [33].

Two cohorts focused on depression-related outcomes. In the UK ROOTS study (n = 603), no prospective associations were detected between a Mediterranean Diet score and later depressive symptoms after robust adjustment for confounders, leading the authors to suggest that previous findings may be due to confounding by lifestyle factors like smoking and alcohol [26]. In contrast, in the Australian Raine cohort (n = 843), a ‘Western’ pattern at 14 years predicted higher BMI and inflammatory markers (leptin, hs-CRP) at 17, which in turn predicted higher depressive symptoms (BDI-Y) and internalising/externalising problems (YSR) [34]. Behavioural outcomes were further examined in the Raine cohort by Trapp et al. [35], linking a ‘Western’ pattern at 14 years to higher externalising problems at 17 in females only.

In a large Canadian cohort (COMPASS; n = 13,887), Dabravolskaj et al. [36] examined specific dietary indicators (fruit and vegetables [F&V], sugar-sweetened beverages [SSBs], junk food, breakfast frequency) in relation to depressive symptoms (CESD-R-10), anxiety symptoms (GAD-7), and psychological well-being (Flourishing Scale) over one year. After adjusting for baseline mental health and numerous covariates, higher baseline SSB consumption predicted greater depressive (β = 0.04) and anxiety symptoms (β = 0.02) and lower psychological well-being (β = −0.03) at follow-up. Higher baseline F&V consumption predicted greater psychological well-being (β = 0.06) but was not significantly associated with depressive or anxiety symptoms after full adjustment. Associations for SSBs were noted to be stronger in males.

Overall, while several cohorts suggest a pattern where poorer diet quality is associated with greater mental health problems [32,33,34,35,36], this finding was not universal. One large UK study found no prospective association after robustly adjusting for confounders [26]. Interpretation across all studies is constrained by attrition, reliance on self-reported diet, and attenuation of effects after adjustment for lifestyle behaviours, suggesting that diet quality may act as part of a broader constellation of health behaviours rather than as an independent causal factor.

### 3.4. Risk of Bias Assessment

The risk of bias across the six included RCTs was varied. Using the Cochrane RoB 2 tool, three trials were rated as having a low risk of bias, one had ‘some concerns’, and two were rated as high risk of bias. High-risk ratings were primarily driven by concerns about missing outcome data (attrition) and deviations from the intended intervention (e.g., poor adherence). The thirteen prospective cohort studies were generally of low-to-moderate quality. Based on the Joanna Briggs Institute (JBI) checklist, eight studies were rated as having a low risk of bias, and five were rated as having a moderate risk. The most common methodological limitation across nearly all cohorts was incomplete follow-up, with many studies not fully describing or exploring the reasons for participant attrition. Detailed domain-level judgements for all included studies are available in the Supplementary Materials (Table S1 and S2).

## 4. Discussion

This systematic review synthesised evidence from six RCTs and thirteen prospective cohort studies examining the relationship between diet and adolescent mental health. Across the RCTs, the evidence was inconsistent: vitamin D supplementation showed mixed effects, a single polyphenol-rich intervention showed a benefit for depressive symptoms, whereas trials of omega-3 fatty acids yielded null findings for mood outcomes. The prospective studies, while heterogeneous, generally supported associations between healthier dietary patterns and better mental health outcomes, particularly fewer depressive symptoms. Together, these findings suggest that diet may play a meaningful role in adolescent mental health while highlighting both the potential of nutritional approaches and the need for more rigorous, standardised research to strengthen the evidence base.

### 4.1. Randomised Controlled Trials

Across the six identified RCTs, the evidence for the benefits of nutrient supplementation on adolescent mental health was inconsistent. For vitamin D and polyphenols, the findings were mixed, with some trials reporting a reduction in depressive symptoms. In contrast, trials of omega-3 fatty acids yielded null findings for mood outcomes. These inconsistencies reflect the small number of trials, heterogeneous methodologies, and varying outcome measures. Assessment tools ranged from validated depression scales such as the CES-D to broader instruments that included mood as a secondary component, such as the Cognitive Drug Research battery, limiting direct comparability.

Intervention modalities also varied widely, spanning vitamin D [18,19,20], omega-3s [21,22], and polyphenol-rich extracts [23]. Differences in dosage, duration, and the baseline nutrient status of participants likely contributed to the varied outcomes. A key limitation across the field was the inconsistent use of biomarkers to verify nutrient uptake. The study by van der Wurff et al. [21] demonstrated the value of this approach, as its biomarker data (Omega-3 Index) revealed that the intervention’s null effect was likely due to poor participant adherence and a failure to reach the target blood levels. Furthermore, while some trials had strong retention, attrition was a significant limitation in several others, with rates exceeding 20%. Sample sizes also ranged from as few as 50 participants [18] to over 450 [20], resulting in marked variation in statistical power. Indeed, adequate statistical power is an important consideration for the RCTs reviewed, as some studies employed relatively small sample sizes (Table 1), making it difficult to distinguish genuine null effects from results generated by underpowered designs.

In sum, most of the identified RCTs (four of six; Table 1) focused on depressive symptoms. This likely reflects the high public health burden of adolescent depression and the availability of validated measurement tools, but it means that potential links between diet and other outcomes such as anxiety, stress regulation, and behavioural problems remain comparatively underexplored. Our risk of bias assessment found two of the six trials to be at high risk of bias. Therefore, the positive findings reported in some studies should be interpreted with significant caution.

### 4.2. Prospective Studies

Thirteen prospective studies explored a wide range of dietary exposures and mental health outcomes. Cohorts generally supported links between healthier diets and fewer depressive symptoms, but the results were inconsistent and often attenuated after adjustment for confounders. Dietary assessment tools varied, from custom-made diet quality scores [33] to narrower adolescent-focused measures like the KIDMED [25] or the Krece Plus [24]. Mental health measures were similarly diverse; for example, depression was captured by use of the BDI-Y [28] and the short form of the MFQ [30] contrasted with broader tools such as the SDQ [32] or less directly relevant scales such as self-efficacy [25].

While most studies relied on self-reported diet, an uncovered issue was where some studies assessed dietary intake at only one time point (e.g., [27]), limiting inference about dietary changes over time. Second, follow-up intervals ranged from less than a year (e.g., [27]) to two years or more (e.g., [32]), with mismatched timings complicating attribution. High attrition was common (e.g., Swann et al. [28] > 50% attrition rate), reducing statistical power and risking bias. Generalisability also varied; while some cohorts were large but demographically narrow, e.g., predominantly White Australian samples [33], others focused on specific groups such as elite Swiss athletes [27], constraining applicability across broader and more general populations.

Although associations sometimes weakened after covariate adjustment (e.g., [26]), the repeated observation that healthier dietary patterns predicted fewer depressive symptoms or better well-being across diverse cohorts [32,33,34,36] highlights a promising avenue that warrants more rigorous investigation. These studies all operationalised diet using whole-diet indices or dietary pattern approaches (e.g., Healthy/Western dietary patterns, diet quality scores, or Mediterranean diet indices) rather than single nutrient exposures. Whole-diet indices may better capture the synergistic effects of multiple nutrients and eating behaviours, helping to explain their more consistent associations compared with single nutrient measures. Nonetheless, differences in dietary assessment methods may make effects non-comparable (e.g., factor analysis-based patterns vs. Mediterranean diet scores).

#### 4.2.1. The Influence of Sex and Socioeconomic Status (SES)

A critical finding from this review is that the relationship between diet and adolescent mental health is not uniform and is likely influenced by demographic and social factors, particularly sex and SES.

#### 4.2.2. Sex-Specific Associations

The potential for sex-specific differences in the diet–mental health relationship is an important but underexplored theme. While many included studies did not report sex-stratified analyses or test for interactions by sex, some did explore these differences, yielding varied results. For instance, in some cohort studies, significant associations were found exclusively in females: Trapp et al. [35] found that a ‘Western’ dietary pattern prospectively predicted greater externalising behaviours only in females. Similarly, Aparicio et al. [24] reported that the link between emotional symptoms and poorer diet quality was present only in females. This suggests that adolescent girls may be more vulnerable to the mental health effects of a poor diet. However, this finding was not universal. One well-controlled study that stratified its analysis found no significant associations in either sex after full adjustment [26]. Furthermore, Dabravolskaj, et al [36] found that the prospective association between higher sugar-sweetened beverage (SSB) consumption and greater depressive and anxiety symptoms was stronger in males. The association between fruit/vegetable intake and well-being did not significantly differ by sex in their study. Future research must prioritise testing for sex differences using interaction analyses to clarify these complex relationships.

#### 4.2.3. Socioeconomic Status

Nearly all the prospective studies adjusted for SES, but they did so using a wide variety of measures, including parental education, household income, and area-level deprivation indices. The effect of this adjustment was not straightforward, revealing the complex role SES plays in the diet–mental health nexus. Our synthesis identified three distinct patterns. (1) Attenuation of effects: In some of the most rigorously controlled studies, adjusting for SES and related lifestyle factors was crucial in attenuating or eliminating the prospective link between diet and mental health [26,32]. This suggests that dietary habits may be part of a broader cluster of socially patterned behaviours and that SES is a powerful confounder. (2) Strengthening or revealing effects: In other studies, the key findings only became statistically significant after adjusting for SES and other covariates [29,35]. This implies that SES can also suppress underlying relationships, and that controlling for it is necessary to isolate the independent effect of diet. (3) Robustness to adjustment: In many studies, the diet–mental health association remained significant even after adjusting for SES [24,25,30,31,33,34]. This provides the strongest evidence for a more direct, independent relationship between what adolescents eat and their psychological well-being. Taken together, these findings show that SES is more than just a simple variable to “control for”. Its inconsistent measurement and complex interplay with diet and mental health is a critical finding and a vital area for future research.

### 4.3. General Methodological Considerations

Integrating the findings from both the RCTs and the prospective cohorts revealed a critical pattern. A key discrepancy emerged: the RCTs, which primarily tested isolated nutrient supplementation (vitamin D and omega-3s), yielded “inconsistent” or “null” findings for mood. In sharp contrast, the prospective studies, which examined whole-diet quality and pattern adherence (e.g., ‘Western’ vs. ‘Healthy’ patterns), “generally supported” an association with mental health outcomes, particularly depressive symptoms.

This discrepancy does not necessarily refute a diet–mental health link. Instead, it may illuminate a key methodological point that aligns with biological plausibility: a ‘recurring pattern’ in the literature is that whole-diet approaches are more consistently associated with mental health than single-nutrient interventions. This suggests that the null effects in the RCTs may stem from methodological factors (such as the poor adherence and failure to reach O-3I targets seen in the van der Wurff et al. [21] trial), or that the synergistic effects of nutrients in whole foods are more impactful than isolated supplements. The consistency of the whole-diet cohort findings, therefore, supports the plausibility of the relationship, even as the RCTs highlight the difficulty of designing an effective supplementation trial.

Taken together, both RCTs and prospective studies suggest that diet may influence adolescent mental health, although methodological challenges limit certainty. RCTs provide stronger causal inference, but remain few, small, and methodologically inconsistent. Prospective studies draw on larger, naturalistic cohorts but are vulnerable to residual confounding, self-report bias, and attrition [37]. Additional heterogeneity arises from the wide range of outcome measures used. Some measures, such as the PedsQL emotional functioning scale [38] and the SDQ emotional symptoms subscale [39], capture broader emotional or internalising difficulties, while others, such as the short MFQ [40], BDI-Y [41], and CES-D [42], focus more specifically on depressive symptomatology. These differences in scope and sensitivity likely contribute to variation in observed associations [43].

A growing body of adolescent research demonstrates high symptom heterogeneity and evolving symptom architecture, with only partial cross-cultural measurement equivalence of common scales and evidence of heterotypic continuity from childhood into adolescence—features that mean that diagnosis-level or composite endpoints can obscure developmentally specific patterns [44,45,46,47]. This aligns with critiques that retrospective harmonisation and top-down diagnostic taxonomies struggle against intrinsic symptom heterogeneity, incompatible instruments, and cohort biases [14,15,48]. Polythetic DSM criteria allow multiple, non-overlapping symptom constellations to share a label; sum-scores can therefore conflate opposing item-level effects. Instruments also differ in purpose and structure—PHQ-9 (DSM-aligned screener), CES-D (population surveillance), HADS-D (medical settings), HAM-D/HDRS, and MADRS (clinician-rated)—and frequently show weak measurement invariance across sex, age, language, country, or time, undermining comparability. Moreover, patient-prioritised domains such as gastrointestinal distress, cognitive difficulties, mental pain, irritability, and emotion regulation are often omitted [49,50]. Therefore, to improve research on the link between nutrition and mental health in adolescents, a symptom-based, dimensional approach is essential. This strategy requires researchers to prespecify primary outcomes, report detailed subscales and key items in addition to total scores, and validate their measurement tools in the relevant population. Furthermore, it is crucial to harmonise core outcome sets across studies and align assessment choices with both the target construct and the priorities of those with lived experience. Employing these practices will reduce outcome misclassification and improve construct validity, making the associations between diet and mental health more interpretable and actionable for adolescents.

Compounding these measurement issues, most studies rely on chronological age instead of pubertal status. Puberty involves significant hormonal shifts (e.g., HPG/HPA axis changes) that reshape mood regulation and increase nutrient needs. Ignoring pubertal timing risks confounding (mistaking puberty’s effects for age or diet effects) and effect modification (as diet’s impact may differ by pubertal stage and sex). Future work must measure pubertal development (e.g., Tanner stage, age at menarche/voice change), incorporate this into analyses (through adjustment or stratification), report sex-specific findings, and ensure that interventions meet the growth-related nutrient demands. Few included studies adequately addressed pubertal status, limiting stage-specific understanding of the diet–mental health link.

Despite these limitations, a recurring pattern emerged—whole-diet quality and pattern adherence are more consistently associated with favourable adolescent mental health outcomes than isolated nutrient supplementation. This likely reflects several factors. Mental health effects may arise from non-additive interactions among co-consumed foods and nutrients; in the future, network approaches (e.g., Gaussian graphical, mutual-information, mixed graphical models) may reveal protective synergies and disruptive clusters that single-nutrient models or simple scores may miss, and recent guidance (Minimal Reporting Standard for Dietary Networks (MRS-DN)) sets reporting standards to improve rigour and comparability [51]. Biologically dietary patterns embody synergistic constellations of foods and nutrients—fibre, polyphenols, unsaturated fats, vitamins, and minerals—that co-modulate interconnected pathways (inflammation, glycaemic/insulin homeostasis, gut–microbiome–brain signalling, neurotrophic support) in a coordinated manner, offering greater biological plausibility than single-nutrient effects [52]. Methodologically, pattern/quality indices implicitly capture the food matrix and its “nutritional dark matter”—the millions of unmeasured bioactives and interactions present in whole foods that single-nutrient studies cannot—while also reducing noise from correlated intakes and self-report error, yielding more stable associations across instruments and samples [51]. Notably, advances in untargeted metabolomics and foodomics are revealing new nutrient candidates with potential mental health relevance—for example, vitamin A5/X (a vegetable- and algae-derived precursor pool for the RXR ligand 9-cis-13,14-dihydroretinoic acid) [53]—further strengthening the rationale for pattern-level approaches.

Beyond methodological considerations, several interpretive insights are noteworthy. Firstly, adolescence may represent a uniquely sensitive period, given rapid brain maturation and developmental transitions that heighten responsiveness to environmental influences [54]. Secondly, the predominance of depressive-symptom outcomes partly reflects the availability of validated tools but may also indicate that depression is particularly nutrition-sensitive in this developmental stage [55]. Thirdly, the weakening of associations after adjustment for covariates indicates that diet is likely embedded within a broader constellation of lifestyle factors rather than acting in isolation [56]. Finally, even modest dietary effects may be meaningful from a public-health perspective, because adolescence is a critical window for the emergence of mental disorders; small improvements in diet at the population level could translate into substantial gains in well-being [57]. However, few studies have simultaneously integrated biological and psychological domains; there is a clear need to identify and validate nutrition-linked biomarkers and correlates of mental health (e.g., inflammatory profiles, glycaemic control, lipid and micronutrient status, microbiome-derived metabolites, and neurotrophic indices, heart rate variability, sleep and other interoceptive indices) [58,59,60] alongside symptom-level outcomes, using longitudinal, multi-informant designs to strengthen inference and translational relevance. A concise roadmap translating these priorities into study designs, measures, biomarkers, and implementation steps is provided in Table 3.

### 4.4. Limitations and Recommendations

The limitations of the current evidence base and the corresponding recommendations for future research are summarised in a detailed roadmap (Table 3). While this agenda is extensive, several priorities are particularly actionable for strengthening the evidence base in the near term. First, future studies should prioritise the use of harmonised, symptom-based outcome measures. As this review demonstrates, the wide range of instruments used in the current literature (e.g., MFQ, BDI-Y, CES-D, PedsQL) is a primary barrier to meta-analysis and cross-study comparison. Second, given our “critical finding” that associations are often attenuated by SES adjustment and may differ by sex, it is essential for future prospective studies to be adequately powered to explicitly test for these interactions. Finally, incorporating biomarker verification (e.g., serum 25(OH)D, Omega-3 Index) is a highly feasible step for intervention trials, as demonstrated by several included studies. This would not only confirm nutrient uptake but also help distinguish true null effects from failures of adherence, a key limitation identified in at least one trial.

Beyond these key priorities, several other limitations warrant consideration. First, applying the WHO definition of adolescence (10–19 years) spans a dynamic developmental period with marked physical, cognitive, and psychosocial change [54]. Such differences may shape responsiveness to dietary exposures. Future work should stratify analyses by developmental stage and employ repeated assessments across adolescence to identify windows of heightened vulnerability or resilience in the diet–mental health relationship.

Second, measurement lacks standardisation. Mental health outcomes have been captured with a wide range of tools—from broader scales such as the SDQ to more targeted measures (e.g., MFQ, BDI-Y, RADS), with the occasional use of constructs less directly aligned to clinical symptoms (e.g., self-efficacy [25]). Dietary exposures likewise vary, from general diet-quality indices based on national guidelines [33] to adolescent-specific measures such as KIDMED and Krece Plus [25]. Greater use of validated, developmentally appropriate tools—and clearer reporting of their comparability—would improve synthesis and interpretation.

Third, the literature remains dominated by depression outcomes, leaving anxiety, stress, externalising behaviours, self-esteem, and aggression comparatively underexplored. Expanding outcome scope and incorporating biomarkers (e.g., inflammatory profiles, glycaemic regulation, micronutrient status, microbiome-derived metabolites, neurotrophic indices) and repeated dietary assessments to complement self-report would strengthen robustness and comparability. Longer follow-up, more frequent measurement, and advanced longitudinal models (e.g., cross-lagged, structural equation modelling) are needed to clarify causal and bidirectional pathways. Furthermore, the certainty of existing evidence is weakened by key methodological issues. As noted in our risk of bias assessment, incomplete follow-up was a near-universal issue across the cohort studies, increasing the risk of attrition bias and limiting the generalisability of the findings.

Finally, unmeasured contextual factors may contribute to observed associations. Few studies adequately considered parental mental health, family dietary culture, or psychosocial stressors, despite their likely influence on both diet and mental health [61]. More diverse, representative adolescent samples are needed, moving beyond homogeneous or niche groups to include under-resourced and high-risk populations. Quasi-experimental approaches (e.g., school- or community-based interventions) can complement RCTs by enhancing external validity and implementation insight; school-based studies have also demonstrated excellent retention, supporting feasibility for adolescent research [22]. While all selected studies were prospective designs intended to reduce the risk of reverse causality, it is important to note that the possibility of residual confounding remains a limitation inherent to all observational data.

Addressing these methodological, conceptual, and developmental gaps would strengthen the evidence base, support more targeted interventions, and enhance translation to clinical and public-health practice [62]. A major strength of the present review is that it did not confine outcomes to diagnostic categories or composite totals; by synthesising evidence across dimensional symptom domains (e.g., depressive and anxiety symptoms, well-being, internalising/externalising), it offers a developmentally sensitive appraisal that is more faithful to adolescent mental-health heterogeneity and more actionable for prevention and intervention.

## 5. Conclusions

Current evidence suggests a possible link between adolescent diet and mental health, albeit with certainty constrained by methodological inconsistency. The most consistent finding across both randomised trials and prospective cohorts is that associations are more reliably observed for whole-diet quality and pattern adherence than for isolated nutrient supplementation. This pattern is biologically plausible and consonant with real-world eating behaviours, indicating that whole-diet approaches are a particularly promising avenue for prevention and intervention, though they require more rigorous testing.

Meaningful progress, however, will depend on addressing key methodological gaps. Future studies must prioritise methodological standardisation—specifically, the use of harmonised, symptom-based, dimensional outcomes rather than diagnostic composites—to improve construct validity and enable cross-study comparison. Furthermore, it is essential to use larger, more diverse samples to explicitly investigate how these associations differ by sex and socioeconomic status (SES), which this review identified as critical and complex factors.

Finally, to strengthen causal inference, research must move beyond observation to integrate mechanistic perspectives. This includes examining inflammatory signalling, gut–brain pathways, and micronutrient status alongside validated biomarkers. Addressing these priorities, as detailed in the research agenda (Table 3), can move the field from tentative associations towards actionable, nutrition-informed strategies that support adolescent mental health.

## Figures and Tables

**Table 1:** Characteristics and Key Findings of Included Randomised Controlled Trials.

Author (Year)	Country	Sub-Category	Dose & Duration	Sample Size (N)	Sample Characteristics	Measures	Sex-Specific Analysis?	SES/Deprivation Adjusted?	Key Findings	Biomarker Verified?
Fisk et al. (2020) [23]	UK	Polyphenols	~253 mg anthocyanins/day for 4 weeks	64	12–17 y, Mixed sex	MFQ, RCADS, PANAS	No	NR	↓ Depressive symptoms NS Anxiety & Affect	No
Grung et al. (2017) [18]	Norway	Vitamin D	1520 IU/day for ~3 months	50	13–14 y, Mixed sex	YSR-CBCL, ToH, ToL	No	NR	NS Internalising/Externalising ↑ Executive function (ToH)	Yes (25(OH)D)
Isaac et al. (2019) [19]	India	Vitamin D	1000 IU/day for 12 weeks	71	11–16 y, Mixed sex, Vit D deficient	WHOQOL-BREF, DASS-21	No	NR	NS Quality of Life NS Anxiety/Stress	Yes (25(OH)D)
Kennedy et al. (2009) [22]	UK	Omega-3	400 or 1000 mg/day (DHA) for 8 weeks	90	10–12 y, Healthy, Mixed sex	CDR battery, VAS mood	No	NR	NS Mood & Cognition	No
Satyanarayana et al. (2024) [20]	India	Vitamin D	~2250 IU/day for 9 weeks	451	14–19 y, Rural, Mixed sex	BDI-II	No	NR	↓ Depressive symptoms	Yes (25(OH)D)
van der Wurff et al. (2020) [21]	Netherlands	Omega-3	400→800 mg/day (EPA + DHA) for 12 months	256	14–15 y, Low O3I, Mixed sex	CES-D, RSE	No	Yes (parental education). Did not affect results.	NS Depressive symptoms NS Self-esteem	Yes (O3I)

Symbols: ↑: Higher or increased levels of exposure/outcome; ↓: Lower or decreased levels of exposure/outcome; →: Associated with or predicts. BDI-II: Beck Depression Inventory-II; CDR: Cognitive Drug Research; CES-D: Centre for Epidemiologic Studies Depression Scale; DASS-21: Depression, Anxiety and Stress Scales-21; DHA: Docosahexaenoic acid; EPA: Eicosapentaenoic acid; IU: International Unit; MFQ: Mood and Feelings Questionnaire; NR: Not Reported; NS: Not Significant; O3I: Omega-3 Index; PANAS: Positive and Negative Affect Schedule; RCADS: Revised Child Anxiety and Depression Scale; RSE: Rosenberg Self-Esteem Scale; ToH: Tower of Hanoi; ToL: Tower of London; VAS: Visual Analogue Scale; Vit D: Vitamin D; WHOQOL-BREF: World Health Organisation Quality of Life-BREF; y: years; YSR-CBCL: Youth Self-Report-Child Behaviour Checklist; 25(OH)D: 25-hydroxyvitamin D.

**Table 2:** Characteristics and Key Findings of Included Prospective Studies.

Author (Year)	Country	Sub-Category	Dietary Exposure & Follow-Up	Sample Size (N)	Sample Characteristics	Measures	Sex-Specific Analysis?	SES/Deprivation Adjusted?	Key Findings
Aparicio et al. (2017) [24]	Spain	Mediterranean Diet	MD adherence & patterns; 3-year follow-up	165	~13.5 y, School cohort	SCARED, CDI, YI-4	Yes. Emotional symptoms linked to poorer diet in females only.	Yes (Hollingshead index). Association remained significant after adjustment; SES was also a predictor.	↑ Emotional symptoms → ↓ MD adherence (Females only, reverse causality tested).
Black et al. (2015) [29]	Australia	Micronutrients	Zinc & Magnesium intake; 3-year follow-up	684	14 & 17 y, General pop.	YSR	No (Interactions tested, NS).	Yes (Family income). Association was significant only after adjustment.	↑ Magnesium → ↓ Externalising problems.
Dabravolskaj et al. (2024) [36]	Canada	Overall Diet Quality	Frequency of fruit/veg, SSB, junk food, breakfast intake; 1-year follow-up	13,887	Adolescents (grades 9–12); mean age 15 y; 52% female; general pop.	Diet: COMPASS survey items	Yes. SSB associations stronger in males.	Yes (Adjusted for weekly spending money, plus lifestyle & psychosocial factors).	↑ SSB intake → ↑ Depressive (β = 0.04) & Anxiety symptoms (β = 0.02), ↓ Well-being (β = −0.03). ↑ F&V intake → ↑ Well-being (β = 0.06), NS Depressive/Anxiety symptoms (after full adjustment).
Gerber et al. (2023) [27]	Switzerland	Macronutrients	Protein intake from food recall; 10-month follow-up	79	~16.4 y, Elite athletes	PHQ-9	No.	No (Measured but excluded from the final model as it was not a significant predictor).	↑ Protein → ↓ Depressive symptoms.
Hayek et al. (2021) [25]	Lebanon	Mediterranean Diet	MD adherence (KIDMED); 1-year follow-up	563	15–18 y, School cohort	Academic Grade	No.	Yes (Parental education). Association remained significant after adjustment.	↑ MD Adherence → ↑ Academic Achievement (Not MH symptoms).
Jacka et al. (2013) [32]	UK	Overall Diet Quality	Healthy vs. unhealthy diet; 2-year follow-up	2383	11–14 y, Socially deprived	SDQ, SMFQ	No (Interactions tested, NS).	Yes (e.g., free school meals). Overall adjustment attenuated the prospective link.	↑ Unhealthy diet → ↑ MH problems (effect attenuated after full adjustment).
Jacka et al. (2011) [33]	Australia	Overall Diet Quality	Healthful vs. unhealthful patterns; 2-year follow-up	2915	11–18 y, General pop.	PedsQL	No (Interactions tested, NS).	Yes (SEIFA area index). Associations remained significant after adjustment.	↑ Healthy diet → ↑ Emotional functioning ↑ Unhealthy diet → ↓ Emotional functioning.
Mrug et al. (2021) [31]	USA	Soft Drinks	Soft drink frequency; 5-year follow-up (3 waves)	5147	11, 13 & 16 y, Diverse pop.	Aggression/Depression Scales	No.	Yes (Education & income). Associations remained significant after adjustment.	↑ Soft Drinks → ↑ Aggression (bidirectional link found)
Oddy et al. (2018) [34]	Australia	Overall Diet Quality	Western’ vs. ‘Healthy’ patterns; 3-year follow-up	843	14 & 17 y, General pop.	BDI-Y, YSR	No (Interactions tested, NS).	Yes (Income & education). Associations remained significant after adjustment.	↑ Western diet → ↑ Depressive symptoms & MH problems (mediated by inflammation).
Swann et al. (2021) [28]	Australia	Macronutrients	Dietary fibre intake; 3-year follow-up	1260	14 & 17 y, General pop.	BDI-Y	No (Interactions tested, NS).	Yes (Education & income). Association was significant after SES adjustment but was attenuated by overall dietary pattern.	↑ Fibre → ↓ Depressive symptoms (attenuated by overall diet quality).
Tolppanen et al. (2012) [30]	UK	Micronutrients	Serum Vitamin D_3_ (biomarker); ~4-year follow-up	2752	~10 to ~14 y, General pop.	MFQ	No (Interactions tested, NS).	Yes (Occupation & education). Associations remained significant after adjustment.	↑ Vitamin D_3_ → ↓ Depressive symptoms.
Trapp et al. (2016) [35]	Australia	Overall Diet Quality	Healthy’ vs. ‘Western’ patterns; 3-year follow-up	746	14 & 17 y, General pop.	YSR	Yes. Link between ‘Western’ diet & externalising found in females only.	Yes (Income & education). Association was significant only after full adjustment.	↑ Western diet → ↑ Externalising problems (females only).
Winpenny et al. (2018) [26]	UK	Mediterranean Diet	MD score from diet diary; 3-year follow-up	603	14.5 & 17.5 y, General pop.	MFQ	Yes. Stratified analysis; no significant associations found in either sex.	Yes (Postcode index). Associations became non-significant after full adjustment.	NS MD Adherence → Depressive symptoms (after full adjustment).

Symbols: ↑: Higher or increased levels of exposure/outcome; ↓: Lower or decreased levels of exposure/outcome; →: Associated with or predicts. BDI-Y: Beck Depression Inventory for Youth; CDI: Children’s Depression Inventory; KIDMED: Mediterranean Diet Quality Index (for children and adolescents); MD: Mediterranean Diet; MFQ: Mood and Feelings Questionnaire; MH: Mental Health; NS: Not Significant; PedsQL: Paediatric Quality of Life Inventory; PHQ-9: Patient Health Questionnaire-9; SCARED: Screen for Child Anxiety Related Disorders; SDQ: Strengths and Difficulties Questionnaire; SEIFA: Socio-Economic Indexes for Areas; SES: Socioeconomic Status; SMFQ: Short Mood and Feelings Questionnaire; y: years; YI-4: Youth Inventory-4; YSR: Youth Self-Report.

**Table 3:** Roadmap for future directions in adolescent nutrition–mental health research.

Priority Area	Significance	Design Implications	Measurement & Biomarkers	Equity & Implementation	Example Research Question
Symptom-based outcomes (beyond diagnoses)	Captures heterogeneity; avoids masking effects in composite totals	Pre-specify primary symptom domains; analyse subscales/items alongside total using appropriate statistical methods	Check that measures work the same across groups (measurement invariance); use multiple informants (young person, parent/carer, teacher, clinician)	Use culturally adapted, validated tools developed with young people	Does improving diet quality reduce anhedonia or irritability specifically in adolescents?
Harmonised core outcome sets	Enables synthesis and comparison across studies	Build a consensus minimal set via Delphi with youth, carers, clinicians, educators to measure priority outcomes	Map existing tools to prioritised outcomes; Determine which priority outcomes lack adequate, validated measurement tools.; Develop and validate measures specifically designed to fill those identified gaps	Co-produce outcomes that matter in schools, clinics and everyday life	What minimal battery best balances burden and validity across settings?
Better exposure assessment	Reduces misclassification of diet	Combine repeated 24 h recalls/diaries with food-frequency tools; include calibration subsamples	Add objective markers: vitamin D (25-hydroxyvitamin D), omega-3 index, carotenoids; digital capture (meal photos, receipts)	Choose methods feasible in low-resource schools/ communities	How do repeated objective diet measures change effect estimates versus food-frequency tools alone?
Biomarker-informed trials	Strengthens causal inference	Target adolescents with low baseline status; stratify randomisation; ensure adequate duration and contrast	Verify exposure change (e.g., vitamin D and omega-3 indices); include inflammation, glycaemic control, neurotrophic factors, microbiome/metabolites	Low-burden sampling (dried blood spots; postal stool kits)	Do adolescents with low vitamin D show greater mood benefit from diet improvement than replete peers?
Dietary network methods (MRS-DN)	Tests synergy rather than single nutrients	Pre-register network models; compare with traditional indices	Report estimation, stability, and sensitivity per the checklist	Build analyst capacity; share code and data	Which food–nutrient constellations most robustly predict symptom improvement?
Longitudinal causal modelling	Clarifies direction of effects	Use within-person change or cross-lagged models; emulate target trials when randomisation is infeasible	Align timing of diet and outcome waves	Plan for retention; minimise burden	Do changes in sugar-sweetened drink intake precede changes in externalising behaviours within individuals?
Mechanistic integration	Links biology to symptoms	Embed mechanistic sub-studies in trials and cohorts	Panels for inflammation, glycaemic control, lipidomics, metabolomics, microbiome, neurotrophic markers	Use affordable, standardised panels; control for batch effects	Are mood benefits mediated by reductions in systemic inflammation?
Early life to adolescence	Tests timing and sensitive periods	Link infancy diet to adolescent outcomes with repeated measures	Include stable markers (e.g., ferritin), growth, and diet trajectories	Maintain diverse cohorts; consider attrition bias	Do infancy diet trajectories interact with adolescent diet to influence mood?
Multi-level context	Accounts for confounding and leverage points	Measure family, school, neighbourhood food environments	Include mealtime practices, food insecurity, marketing exposure	Oversample under-resourced and high-risk groups	Does family meal frequency change the effect of diet quality on anxiety?
Sex & Gender Analysis	Addresses findings that diet-mental health links may be stronger or exclusive to females; clarifies vulnerability.	Power studies adequately to test for sex/gender interactions; pre-specify stratified analyses rather than adding them post hoc	Collect data on sex assigned at birth and gender identity; consider hormonal status/pubertal stage as a potential moderator	Ensure interventions and recommendations are relevant and communicated effectively to adolescents of all genders	Is the prospective link between a ‘Western’ diet and externalising behaviours stronger in adolescent girls than in boys?
Socioeconomic & Environmental Context	Disentangles the independent effect of diet from the powerful confounding effects of deprivation, stress, and food insecurity.	Use robust, multi-domain SES measures (parental income, education, area-level deprivation); measure the food environment (e.g., food deserts)	Validated SES indices (e.g., SEIFA); household food insecurity scales; geospatial data on local food outlets	Oversample low-SES and high-risk groups; co-design interventions that are affordable, accessible, and culturally appropriate	Does the effect of a healthy diet on depressive symptoms persist after fully accounting for family income and food insecurity?
Implementation and scale-up	Ensures real-world impact	Hybrid effectiveness-implementation designs	Track fidelity, reach, cost, acceptability, and equity metrics	Co-design with schools and commissioners	What is the cost per meaningful gain in wellbeing for a school diet programme?
Open science and standardisation	Improves trust and synthesis	Register protocols; share data and code; use reporting checklists	Common data dictionaries; transparent preprocessing	Capacity building and training	Does pre-registration reduce outcome-reporting bias in diet–mental health trials?

## Data Availability

Data are available at https://osf.io/c6xze (accessed on 20 October 2025).

## Supplementary Materials

The following supporting information can be downloaded at: https://www.mdpi.com/article/10.3390/nu17233677/s1, Table S1: Cochrane Risk of Bias 2 assessment of randomised controlled trials; Table S2: Joanna Briggs Institute Critical Appraisal for Prospective Studies; PRISMA Checklists S1a,b and S2.
