# Supplementary material for: A Recipe for Resilience: A Systematic Review of Diet and Adolescent Mental Health
Source: Nutrients. 2025 Nov 24;17(23):3677. doi: 10.3390/nu17233677 (PMC12693682; doi:10.3390/nu17233677)
Supplement: Supplementary file 1 [file nutrients-17-03677-s001.zip › nutrients-3985605-supplementary.pdf]

Supplementary Materials

The following are available:

Table S1: Cochrane Risk of Bias 2 assessment of randomised controlled trials,

Table S2: Joanna Briggs Institute Critical Appraisal for Prospective Studies.

As well as the PRISMA checklists (PRISMA checklist S1a and PRISMA checklist S1b), and the Joanna Briggs Institute Critical Appraisal Tool Checklist (Checklist S2)

Table S1. Cochrane Risk of Bias 2 assessment of randomised controlled trials – Algorithm Results

Author (year)	Randomisation process	Deviations from intended interventions	Missing outcome data	Measurement of the outcome	Selection of the reported result	Overall
Fisk et al. (2020)	Low risk (+)	Low risk (+)	Low risk (+)	Low risk (+)	Low risk (+)	Low risk (+)
Grung et al. (2017)	Low risk (+)	Low risk (+)	Some concerns (!)	Low risk (+)	Low risk (+)	Some concerns (!)
Isaac et al. (2019)	Low risk (+)	Low risk (+)	Low risk (+)	Low risk (+)	Low risk (+)	Low risk (+)
Kennedy et al. (2009)	Low risk (+)	Low risk (+)	Low risk (+)	Low risk (+)	Low risk (+)	Low risk (+)
Satyanarayana et al. (2024)	Low risk (+)	Low risk (+)	High risk (-)	Low risk (+)	Low risk (+)	High risk (-)
van der Wurff et al. (2020)	Low risk (+)	High risk (-)	High risk (-)	Low risk (+)	Low risk (+)	High risk (-)

	Note. '+' = low risk of bias, '-'= high risk of bias, and '!' = some concerns of bias.
	**Table S2. Joanna Briggs Institute Critical Appraisal for Prospective Studies**

Author	***1***	***2***	***3***	***4***	***5***	***6***	***7***	***8a***	***8b***	***9***	***10***	***Overall risk of Bias***
Aparicio et al. (2017)	yes	yes	yes	yes	yes	no	yes	no	no	no	yes	Moderate
Black et al. (2015)	yes	yes	yes	yes	yes	no	yes	no	yes	yes	yes	Low
Gerber et al. (2023)	yes	yes	yes	yes	yes	no	yes	no	no	no	yes	Moderate
Hayek et al. (2021)	yes	yes	yes	yes	yes	yes	yes	no	no	yes	yes	Moderate
Jacka et al. (2011)	yes	yes	yes	yes	yes	yes	yes	no	yes	yes	yes	Low
Jacka et al. (2013)	yes	yes	yes	yes	yes	yes	yes	no	yes	yes	yes	Low
Mrug et al. (2021)	yes	yes	yes	yes	yes	yes	yes	no	yes	yes	yes	Low
Oddy et al. (2018)	yes	yes	yes	yes	yes	yes	yes	no	yes	yes	yes	Low
Swann et al. (2021)	yes	yes	yes	yes	yes	yes	yes	no	no	yes	yes	Moderate
Trapp et al. (2016)	yes	yes	yes	yes	yes	yes	yes	no	yes	yes	yes	Low
Winpenny et al. (2018)	yes	yes	yes	yes	yes	yes	yes	no	yes	yes	yes	Low

Tolppanen et al. (2012)	yes	yes	yes	yes	yes	yes	yes	no	no	yes	yes	Moderate
Dabravolskaj et al., 2024	yes	yes	yes	yes	yes	yes	yes	yes	yes	no	yes	Low
	Low risk of bias: *>70% 'yes' answers*<br>***Moderate risk of bias:*** *50%–69% 'yes' answers*<br>***High risk of bias:*** *<50% 'yes' answers*										

PRISMA Checklist S1a.

- Section and Topic	Item #	Checklist item	Reported (Yes/No)
**TITLE**			
Title	1	Identify the report as a systematic review.	yes
**BACKGROUND**			
Objectives	2	Provide an explicit statement of the main objective(s) or question(s) the review addresses.	yes
**METHODS**			
Eligibility criteria	3	Specify the inclusion and exclusion criteria for the review.	yes
Information sources	4	Specify the information sources (e.g. databases, registers) used to identify studies and the date when each was last searched.	yes
Risk of bias	5	Specify the methods used to assess risk of bias in the included studies.	yes
Synthesis of results	6	Specify the methods used to present and synthesise results.	yes
**RESULTS**			
Included studies	7	Give the total number of included studies and participants and summarise relevant characteristics of studies.	yes
Synthesis of results	8	Present results for main outcomes, preferably indicating the number of included studies and participants for each. If meta-analysis was done, report the summary estimate and confidence/credible interval.	yes

PRISMA Checklist S1a.			
- **Section and Topic**	**Item #**	**Checklist item**	**Reported (Yes/No)**
		If comparing groups, indicate the direction of the effect (i.e. which group is favoured).	
**DISCUSSION**			
Limitations of evidence	9	Provide a brief summary of the limitations of the evidence included in the review (e.g. study risk of bias, inconsistency and imprecision).	yes
Interpretation	10	Provide a general interpretation of the results and important implications.	yes
**OTHER**			
Funding	11	Specify the primary source of funding for the review.	yes
Registration	12	Provide the register name and registration number.	yes

Prisma Checklist S1b			
**Section and Topic**	**Item #**	**Checklist item**	**Location where item is reported**
**TITLE**			
Title	1	Identify the report as a systematic review.	Page 1
**ABSTRACT**			
Abstract	2	See the PRISMA 2020 for Abstracts checklist.	Page 1

**Prisma Checklist S1b**			
**Section and Topic**	**Item #**	**Checklist item**	**Location where item is reported**
**INTRODUCTION**			
Rationale	3	Describe the rationale for the review in the context of existing knowledge.	Section 1
Objectives	4	Provide an explicit statement of the objective(s) or question(s) the review addresses.	Section 1
**METHODS**			
Eligibility criteria	5	Specify the inclusion and exclusion criteria for the review and how studies were grouped for the syntheses.	Section 2.2, 2.4, 2.5
Information sources	6	Specify all databases, registers, websites, organisations, reference lists and other sources searched or consulted to identify studies. Specify the date when each source was last searched or consulted.	Section 2.1
Search strategy	7	Present the full search strategies for all databases, registers and websites, including any filters and limits used.	Section 2.1
Selection	8	Specify the methods used to decide whether a study met	Section 2.1

Prisma Checklist S1b			
**Section and Topic**	**Item #**	**Checklist item**	**Location where item is reported**
process		the inclusion criteria of the review, including how many reviewers screened each record and each report retrieved, whether they worked independently, and if applicable, details of automation tools used in the process.	
Data collection process	9	Specify the methods used to collect data from reports, including how many reviewers collected data from each report, whether they worked independently, any processes for obtaining or confirming data from study investigators, and if applicable, details of automation tools used in the process.	Section 2.3
Data items	10a	List and define all outcomes for which data were sought. Specify whether all results that were compatible with each outcome domain in each study were sought (e.g. for all measures, time points, analyses), and if not, the methods used to decide which results to collect.	Section 2.4
	10b	List and define all other variables for which data were sought (e.g. participant and intervention characteristics,	Section 2.3

**Prisma Checklist S1b**			
**Section and Topic**	**Item #**	**Checklist item**	**Location where item is reported**
		funding sources). Describe any assumptions made about any missing or unclear information.	
Study risk of bias assessment	11	Specify the methods used to assess risk of bias in the included studies, including details of the tool(s) used, how many reviewers assessed each study and whether they worked independently, and if applicable, details of automation tools used in the process.	Section 2.6
Effect measures	12	Specify for each outcome the effect measure(s) (e.g. risk ratio, mean difference) used in the synthesis or presentation of results.	N/A
Synthesis methods	13a	Describe the processes used to decide which studies were eligible for each synthesis (e.g. tabulating the study intervention characteristics and comparing against the planned groups for each synthesis (item #5)).	Section 2.1
	13b	Describe any methods required to prepare the data for presentation or synthesis, such as handling of missing summary statistics, or data conversions.	N/A

Prisma Checklist S1b			
**Section and Topic**	**Item #**	**Checklist item**	**Location where item is reported**
	13c	Describe any methods used to tabulate or visually display results of individual studies and syntheses.	Tables
	13d	Describe any methods used to synthesize results and provide a rationale for the choice(s). If meta-analysis was performed, describe the model(s), method(s) to identify the presence and extent of statistical heterogeneity, and software package(s) used.	2.5
	13e	Describe any methods used to explore possible causes of heterogeneity among study results (e.g. subgroup analysis, meta-regression).	N/A
	13f	Describe any sensitivity analyses conducted to assess robustness of the synthesized results.	N/A
Reporting bias assessment	14	Describe any methods used to assess risk of bias due to missing results in a synthesis (arising from reporting biases).	N/A
Certainty	15	Describe any methods used to assess certainty (or	N/A

Prisma Checklist S1b			
**Section and Topic**	**Item #**	**Checklist item**	**Location where item is reported**
assessment		confidence) in the body of evidence for an outcome.	
**RESULTS**			
Study selection	16a	Describe the results of the search and selection process, from the number of records identified in the search to the number of studies included in the review, ideally using a flow diagram.	Section 3.1
	16b	Cite studies that might appear to meet the inclusion criteria, but which were excluded, and explain why they were excluded.	Section 3.1
Study characteristics	17	Cite each included study and present its characteristics.	Sections 3.2, 3.3, Table 1, Table 2
Risk of bias in studies	18	Present assessments of risk of bias for each included study.	Section 3.4 & Supplementary Tables S1, S2
Results of individual	19	For all outcomes, present, for each study: (a) summary statistics for each group (where appropriate) and (b) an	N/A

Prisma Checklist S1b			
**Section and Topic**	**Item #**	**Checklist item**	**Location where item is reported**
studies		effect estimate and its precision (e.g. confidence/credible interval), ideally using structured tables or plots.	
Results of syntheses	20a	For each synthesis, briefly summarise the characteristics and risk of bias among contributing studies.	Section 2.6
	20b	Present results of all statistical syntheses conducted. If meta-analysis was done, present for each the summary estimate and its precision (e.g. confidence/credible interval) and measures of statistical heterogeneity. If comparing groups, describe the direction of the effect.	N/A
	20c	Present results of all investigations of possible causes of heterogeneity among study results.	N/A
	20d	Present results of all sensitivity analyses conducted to assess the robustness of the synthesized results.	N/A
Reporting biases	21	Present assessments of risk of bias due to missing results (arising from reporting biases) for each synthesis assessed.	Section 3.4

Prisma Checklist S1b

Section and Topic	Item #	Checklist item	Location where item is reported
Certainty of evidence	22	Present assessments of certainty (or confidence) in the body of evidence for each outcome assessed.	N/A
**DISCUSSION**			
Discussion	23a	Provide a general interpretation of the results in the context of other evidence.	Section 4.0
	23b	Discuss any limitations of the evidence included in the review.	Section 4.0
	23c	Discuss any limitations of the review processes used.	Section 4.4
	23d	Discuss implications of the results for practice, policy, and future research.	Section 4.4, Table 3, Section 5
**OTHER INFORMATION**			
Registration and protocol	24a	Provide registration information for the review, including register name and registration number, or state that the review was not registered.	Section 2.0
	24b	Indicate where the review protocol can be accessed, or state that a protocol was not prepared.	Section 2

Prisma Checklist S1b			
**Section and Topic**	**Item #**	**Checklist item**	**Location where item is reported**
	24c	Describe and explain any amendments to information provided at registration or in the protocol.	Section 2
Support	25	Describe sources of financial or non-financial support for the review, and the role of the funders or sponsors in the review.	Cover sheet
Competing interests	26	Declare any competing interests of review authors.	Cover sheet
Availability of data, code and other materials	27	Report which of the following are publicly available and where they can be found: template data collection forms; data extracted from included studies; data used for all analyses; analytic code; any other materials used in the review.	Cover sheet

Checklist S2. Joanna Briggs Institute Critical Appraisal Tool Checklist	
**Question #**	**Question**

**1**	*Were the two groups similar and recruited from the same population?*
**2**	*Were the exposures measured similarly to assign people to both exposed and unexposed groups?*
**3**	*Was the exposure measured in a valid and reliable way?*
**4**	*Were confounding factors identified?*
**5**	*Were strategies to deal with confounding factors stated?*
**6**	*Were the groups/ participants free of the outcome at the start of the study (or at the moment of exposure)?*
**7**	*Were the outcomes measured in a valid and reliable way?*
**8**	*Was (A) follow up complete, and if not, were (B) the reasons to loss to follow up described and explored?*
**9**	*Were strategies to address incomplete follow up utilized?*
**10**	*Was an appropriate statistical analysis used?*

SCOPUS search

(TITLE-ABS-KEY (adolescent OR adolescence OR teen*)) AND (TITLE-ABS-KEY ((food OR meal OR diet OR "dietary pattern" OR nutrient) W/8 ("Mental health" OR "Mood" OR "Depression" OR "Well-being" OR "Stress" OR "Anxiety" OR "Self-esteem" OR "Emotional regulation" OR "Behavioural health"))) AND (TITLE-ABS-KEY ("controlled trial" OR "randomized controlled trial" OR RCT OR prospective OR longitudinal OR cohort)) AND (LANGUAGE (english)) AND (PUBYEAR < 2025) AND NOT (DOCTYPE (re)) AND NOT (TITLE-ABS-KEY ("cross-sectional" OR "cross sectional" OR retrospective OR "case control" OR "eating disorder*"))
